# How important is the role of iterative liver direct surgery in patients with hepatocellular carcinoma for a transplant center located in an area with a low rate of deceased donation?

**DOI:** 10.3389/fonc.2022.929607

**Published:** 2022-07-29

**Authors:** Duilio Pagano, Simone Khouzam, Bianca Magro, Marco Barbara, Davide Cintorino, Fabrizio di Francesco, Sergio Li Petri, Pasquale Bonsignore, Sergio Calamia, Giacomo Deiro, Calogero Cammà, Marco Canzonieri, Salvatore Gruttadauria

**Affiliations:** ^1^ Department for the Treatment and Study of Abdominal Diseases and Abdominal Transplantation, IRCCS-ISMETT (Istituto di Ricovero e Cura a Carattere Scientifico-Istituto Mediterraneo per i Trapianti e Terapie ad Alta Specializzazione), UPMC (University of Pittsburgh Medical Center), Palermo, Italy; ^2^ Department of Surgery, Penn State Health Milton S. Hershey Medical Center, Hershey, PA, United States; ^3^ Research Department, IRCCS-ISMETT (Istituto di Ricovero e Cura a Carattere Scientifico - Istituto Mediterraneo per i Trapianti e Terapie ad alta specializzazione), Palermo, Italy; ^4^ Section of Gastroenterology & Hepatology, Department of Health Promotion, Mother and Child Care, Internal Medicine and Medical Specialties, PROMISE, University of Palermo, Palermo, Italy; ^5^ Department of General Surgery and Medical-Surgical Specialties, University of Catania, Catania, Italy

**Keywords:** liver transplantation, laparoscopic, liver resection, hepatocellular carcinoma, thermal ablation

## Abstract

**Introduction:**

Hepatocellular carcinoma (HCC) accounts for nearly 90% of primary liver cancers, with estimates of over 1 million people affected by 2025. We aimed to explore the impacting role of an iterative surgical treatment approach in a cohort of HCC patients within the Milan criteria, associated with clinical risk factors for tumor recurrence (RHCC) after liver transplant (LT) and loco-regional therapies (LRT), as well as liver resection (LR) and/or microwave thermal ablation (MWTA).

**Methods:**

We retrospectively analyzed our experience performed during an 8-year period between January 2013 and December 2021 in patients treated for HCC, focusing on describing the impact on preoperative end-stage liver disease severity, oncologic staging, tumor characteristics, and surgical treatments. The Cox model was used to evaluate variables that could predict relapse risks. Relapse risk curves were calculated according to the Kaplan–Meier method, and the log-rank test was used to compare them.

**Results:**

There were 557 HCC patients treated with a first-line approach of LR and/or LRTs (*n* = 335) or LT (*n* = 222). The median age at initial transplantation was 59 versus 68 for those whose first surgical approach was LR and/or LRT. In univariate analysis with the Cox model, nodule size was the single predictor of recurrence of HCC in the posttreatment setting (HR: 1.61, 95% CI: 1.05–2.47, *p* = 0.030). For the LRT group, we have enlightened the following clinical characteristics as significantly associated with RHCC: hepatitis B virus infection (which has a protective role with HR: 0.34, 95% CI: 0.13–0.94, *p* = 0.038), number of HCC nodules (HR: 1.54, 95% CI: 1.22–1.94, *p* < 0.001), size of the largest nodule (HR: 1.06, 95% CI: 1.01–1.12, *p* = 0.023), serum bilirubin (HR: 1.57, 95% CI: 1.03–2.40, *p* = 0.038), and international normalized ratio (HR: 16.40, 95% CI: 2.30–118.0, *p* = 0.006). Among the overall 111 patients with RHCC in the LRT group, 33 were iteratively treated with further curative treatment (12 were treated with LR, two with MWTA, three with a combined LR-MWTA treatment, and 16 underwent LT). Only one of 18 recurrent patients previously treated with LT underwent LR. For these RHCC patients, multivariable analysis showed the protective roles of LT for primary RHCC after IDLS (HR: 0.06, 95% CI: 0.01–0.36, *p* = 0.002), of the time relapsed between the first and second IDLS treatments (HR: 0.97, 95% CI: 0.94–0.99, *p* = 0.044), and the impact of previous minimally invasive treatment (HR: 0.28, 95% CI: 0.08–1.00, *p* = 0.051).

**Conclusion:**

The coexistence of RHCC with underlying cirrhosis increases the complexity of assessing the net health benefit of ILDS before LT. Minimally invasive surgical therapies and time to HCC relapse should be considered an outcome in randomized clinical trials because they have a relevant impact on tumor-free survival.

## Introduction

Hepatocellular carcinoma (HCC) accounts for nearly 90% of primary liver cancers, with over 1 million people affected by 2025 (1). Since 2018, HCC has remained the sixth most common cancer and the third most fatal cancer globally (2, 3). Liver resection (LR), liver transplant (LT), and thermal ablations are the curative surgical treatment options for HCC, but each option depends on the number of nodules, tumor diameter, vascular invasion, extrahepatic disease, and shortage of deceased donor pool for LT.

The Barcelona Clinic Liver Cancer (BCLC) classification system has been approved by the American Association for the Study of Liver Diseases (AASLD), the American Gastroenterological Association, and the European Association for the Study of the Liver (EASL) to indicate a specific therapeutic option for HCC at each. Resection is recommended for those at BCLC stage 0 or BCLC-A with a solitary nodule (4, 5). Additionally, the patient must be an optimal candidate meeting the following criteria: compensated Child–Pugh class A liver function, model for end-stage liver disease (MELD) score of <10, and matched grade portal hypertension (4, 6, 7). In Asia, the classification system is designed to detect HCC earlier with higher sensitivity and lower specificity (8). The Korean Liver Cancer Association - National Cancer Center revised its guidelines in 2018 to advise the management of early HCC with early treatment, such as local-regional ablations or trans-arterial chemoembolization (TACE) (9, 10). Still, the median OS for treated HCC is ≥60 months, with a 5-year survival rate approaching 70%; HCC recurrence (RHCC) develops in nearly 70% of patients within 5 years after initial resection (1).

LT is the definitive treatment option for HCC patients within the eligible Milan criteria, but LT must be safeguarded with consideration given to LR before LT due to the possibility of recurrence, waiting list times, and limited organ supply (10–13). Of note, the Italian organ allocation system differs from MELD such that it is a blended model of urgency, utility, and transplant benefit (14, 15). Regardless, there remains a high likelihood, 6%–18%, of RHCC, with 40% to 50% occurring within the first year after LT and 20% occurring during the second year (16, 17). Immunosuppressive regimens and surgical decision-making should also consider the fractional allele imbalance as it provides critical information on the risk of HCC recurrence (18). Given the high likelihood of RHCC, consideration of posttransplant recurrence and outcomes must be considered (19, 20).

Well-known predictors of poor prognosis after LR are diameter of ≥5 cm, multiple tumors, no capsular formation, vascular invasion, TNM classification stage 3 or 4, and alpha-fetoprotein (AFP) of at least 32 ng/ml (21–23). Similarly, risk factors for RHCC with open LR followed by LT were determined to be elevated AFP levels, microvascular invasion, tumor grade, and multinodular tumors. However, instead of primary LT or open LR, laparoscopic liver resection (LLR) and ablation before LT are the new preferred treatment approaches due to the significant improvements in survival and patient outcomes (24–27). This study aims to describe our experience in applying the iterative treatment approach to a cohort of HCC patients within the Milan criteria and to explore the role of surgical management and clinical risk factors that could impact RHCC and OS after LT.

## Materials and methods

Here, we report a series of HCC patients’ management and treatments at the Mediterranean Institute for Transplantation and Highly Specialized Therapies (ISMETT) center with the aim of analyzing RHCC in terms of associated risk factors and best treatment options in those treated with iterative liver direct surgery strategy (ILDS) as LR and loco-regional therapy (LRT), during an 8-year period between January 2013 and December 2021. All data were collected using the electronic database and processed retrospectively. The diagnosis of HCC was made in the period before undergoing locoregional procedures, receiving an LR and/or LRT, or being listed for LT, following the criteria of the main AASLD and EASL-EORTC Clinical Practice Guidelines (28, 29). LT included both living and deceased donors, with one donor having died of cardiac death. Patients receiving living-donor LT, however, were not included in the following analysis. The surgical treatment option was selected after a careful multidisciplinary evaluation of the patient and considering staging, tumor location, and residual liver function (30). Before operating, the criteria for judging patients suitable for HCC resection were as follows: (1) BCLC 0/A (no macrovascular invasion or distant/lymphatic metastasis); (2) Child–Pugh grade A/B. Patients who did not meet the LR or LLR criteria were then considered for percutaneous microwave thermal ablation (MWTA). In cases where HCC nodules were challenging to approach percutaneously or in patients with moderated ascites, MWTAs were performed with a laparoscopic approach (LMWTA). The remaining subset of HCC patients underwent a transplant evaluation and were only included in the list after radiological confirmation of compliance with the Milan criteria (single nodule ≤5 cm or up to three nodules each ≤3cm, in the absence of macrovascular infiltration and distant metastases). In some doubtful cases, it was also necessary to perform a biopsy examination.

Patients with a diagnosis of RHCC were only considered for LT if the HCC fell within the Milan criteria and were able to meet the other transplant evaluation criteria. After surgical procedures, all patients underwent a follow-up protocol every 3 months for the first year and twice per year thereafter. The protocol included serum levels of AFP, ultrasonography, abdomen computed tomography (CT) with contrast medium, and/or magnetic resonance imaging (MRI) with hepato-specific contrast medium. In cases of ascertained or suspected recurrence of intrahepatic and/or extrahepatic HCC, other investigations were performed: liver MRI, chest CT, bone scan, ultrasound-guided biopsy, or positron emission tomography (PET). The parameters evaluated in the recruited patients are the number of liver lesions compatible with HCC, the site, the maximum size of the tumor, the presence or absence of angiolymphatic invasion, the execution of previous LRTs, the tumor histologic type, and tumor recurrence. Whenever necessary, patients underwent bridging or downstaging procedures (TACE or percutaneous ablations) in an attempt to maintain the patient’s suitability for LT.

The clinical endpoint of this study was time to RHCC after first-line treatment and time to further RHCC after an iterative treatment. We also evaluated patients’ overall survival after LT.

### Statistical analysis

Patients’ characteristics are summarized as the median and interquartile range (IQR) or as frequency and percentage, as appropriate. Overall survival and time to recurrence were estimated by means of Kaplan–Meier estimators and tested for differences by means of Log-rank tests. Time to recurrence was defined as the number of days between the transplant and the first radiological evidence of tumor recurrence. The proportional hazard (PH) assumption between LR/MWTA group and LT was tested both graphically, through a complementary log–log transformation of survival curves, and parametrically, by testing the slope of Schoenfeld residuals with respect to survival times (Harrell–Lee test). Whenever the PH assumption did not hold, PH Cox models were then stratified with respect to the treatment group. A multivariable model was selected by means of a forward stepwise regression algorithm using the Akaike information criterion as a stopping rule. All hypotheses were tested at *α* = 0.05 significance level. All analyses and graphics were performed using the R statistical environment, version 4.1.2.

## Results

### Study population

**Figure 1:**
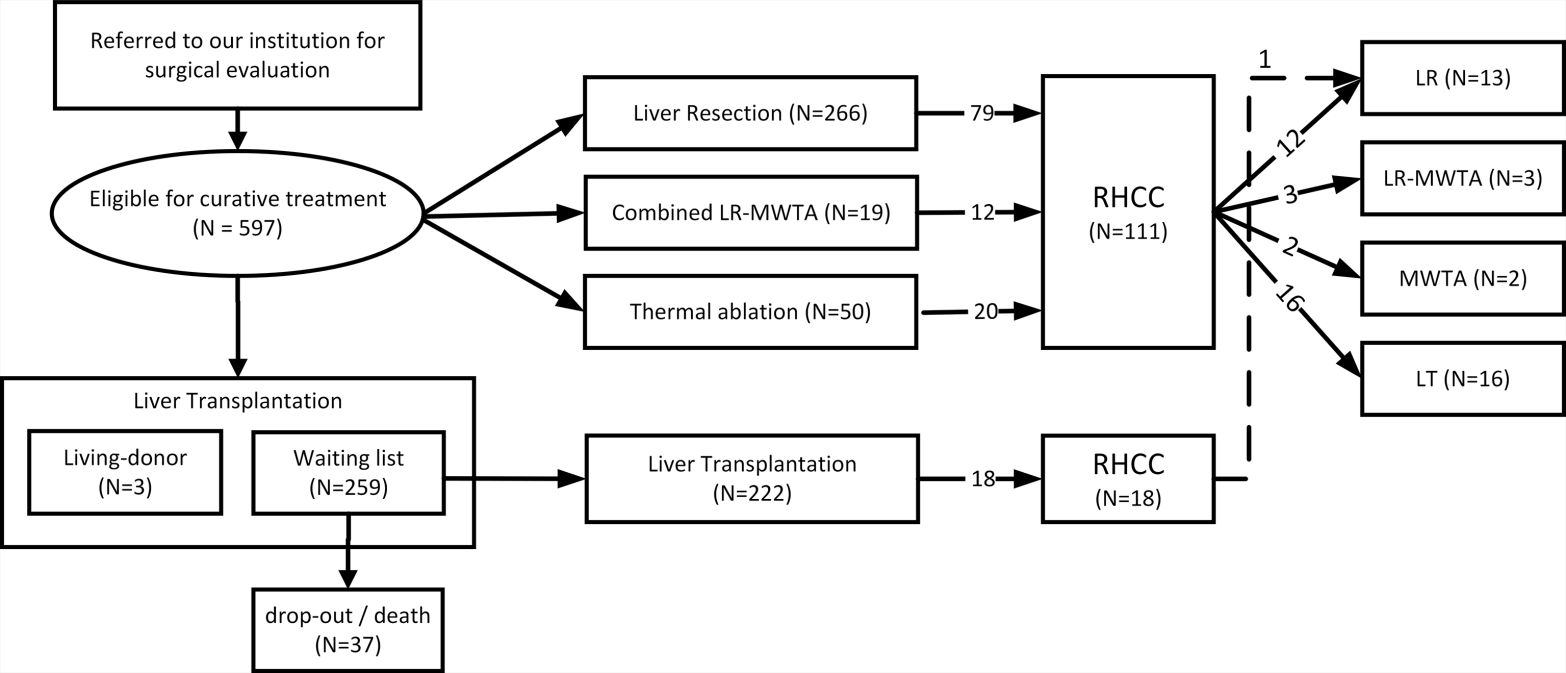
Patient and treatment selection flowchart.

Between January 2013 and December 2021, 597 patients with a recent diagnosis of HCC were judged to be eligible for surgical curative treatment among those referred to our institution for surgical evaluation. Of these, 335 were suitable for LR or thermal ablation, three underwent a living-donor LT, and 259 entered the waiting list for LT (**Figure 1**). Thirty-seven patients were subsequently removed from the waiting list due to disease worsening or death, the remaining 222 underwent a deceased-donor LT. **Table 1** presents the clinical characteristics of 557 patients who were surgically treated for HCC either with a LR and/or MWTA (*N* = 335) or with a deceased-donor LT (*N* = 222).

**Table 1:** Clinical and demographic characteristics of 557 patients affected by hepatocellular carcinoma who underwent surgical treatment.

	Liver resection/ablation	Liver transplantation	Overall
N	**335**	**222**	**557**
Male sex (no. (%))	255 (76)	177 (80)	432 (78)
Age ((years), median [IQR])	68 [61, 73]	59 [53, 64]	
Etiology of liver disease (no. (%))			
Hepatitis C virus-related liver cirrhosis	216 (64)	117 (53)	333 (60)
Hepatitis B virus-related liver cirrhosis	32 (9)	29 (13)	61 (11)
Alcohol-related liver cirrhosis	15 (5)	30 (13)	45 (8)
Nonalcoholic fatty liver disease	59 (18)	42 (19)	101 (18)
Cryptogenic cirrhosis	2 (0.6)	1 (0.5)	3 (0.5)
Noncirrhotic liver	9 (3)	0 (0)	9 (2)
Cholestatic liver disease	2 (0.6)	3 (1.4)	5 (0.9)
Number of HCC lesions (no. (%))			
1	253 (76)	130 (59)	383 (69)
2	63 (19)	52 (23)	115 (21)
3	19 (6)	40 (18)	59 (11)
Maximum tumor size (median [IQR])	3.0 [1.8, 4.6]	2.2 [1.5, 3.2]	2.5 [1.7, 4.0]
Histological size of the tumor (pT)			
T1	162 (48)	90 (41)	252 (45)
T2	127 (38)	123 (55)	250 (45)
T3/T4	46 (14)	9 (4)	55 (10)
Bilirubin ((mg/dl), median [IQR])	0.6 [0.4, 0.9]	1.5 [0.8, 3.0]	0.8 [0.5, 1.4]
INR (median [IQR])	1.1 [1.0, 1.1]	1.2 [1.1, 1.4]	1.1 [1.0, 1.2]
Platelet count ((×10^9^/L), median [IQR])	156 [111, 215]	75 [51, 98]	117 [75, 180]
Creatinine ((mg/dl), median [IQR])	0.9 [0.8, 1.1]	0.8 [0.7, 1.1]	0.9 [0.8, 1.1]
Child–Pugh score			
A5	240 (72)	33 (15)	273 (49)
A6	75 (22)	48 (22)	123 (22)
B7	8 (2)	50 (23)	58 (10)
B8	2 (1)	28 (13)	30 (5)
B9	10 (3)	37 (16)	47 (8)
C10	0 (0)	22 (10)	22 (4)
C11	0 (0)	4 (2)	4 (0.7)
MELD-Na (median [IQR])	9 [8, 10]	13 [10, 17]	10 [8, 13]
Histological grading			
G1	63 (19)	56 (25)	119 (21)
G2	154 (46)	76 (34)	230 (41)
G3	97 (29)	89 (40)	186 (33)
G4	21 (6)	4 (2)	25 (4)
Vascular invasion (no. (%))	112 (33)	43 (19)	155 (28)
First treatment			
Liver resection (LR)	266 (79)	**–**	266 (48)
Microwave thermal ablation (MWTA)	50 (15)	**–**	50 (9)
Combined LR/MWTA	19 (6)	**–**	19 (3)
Liver transplantation (LT)	**–**	222 (100)	222 (40)
Minimally invasive approach (no. (%))	176 (53)	0 (0)	176 (32)
Second treatment	**33 (10)**	**1 (0.5)**	**34 (6)**
LR	12 (3.6)	1 (0.5)	13 (2)
MWTA	2 (0.6)	0 (0)	2 (0.4)
Combined LR/MWTA	3 (0.9)	0 (0)	3 (0.5)
OLT	16 (4.7)	0 (0)	16 (3)
Months between first and second treatment [median (IQR)]	19.9 (7.3–32.9)	0.5 (0.3, 34.7)	19.9 [7.0, 33.9]

The bold values provided information about second treatments, and the following surgical options are the specific treatments: LRMWTACombined LR/MWTAOLT.

The LR/MWTA group accounted for 335 patients; of these, 255 were men (76%), and 80 were women (24%); 266 (79%) were treated with LR, 50 (15%) with MWTA, and 19 (6%) with a combined LR/MWTA treatment. A minimally invasive method of LLR or LMWTA was preferred whenever possible and used 53% of the time. Most of the patients in the LR/MWTA group had very well-compensated liver disease. In particular, 315 (94%) patients were in Child–Pugh class A, 20 (6%) were in class B, and no patients were in Child–Pugh class C.

The median clinical MELD-Na score was 9 (IQR: 8–10) at the time of treatment. On imaging techniques, 253 patients (76%) had monofocal HCC, 63 patients (19%) had bifocal HCC, and 19 patients (6%) had up to three HCC nodules. In addition, histological grading was analyzed according to the Edmonson and Steiner classification (G1: well-differentiated, G2: moderately differentiated, G3: poorly differentiated, G4: undifferentiated) and 65% (*N* = 217) of patients presented with well- or moderately differentiated HCC, while 35% (*N* = 118) had a poorly differentiated HCC. Tumor sizes were histologically classified (pT) as T1 in 162 cases (48%), T2 in 127 cases (38%), and T3 in the remaining 46 (14%). The microvascular invasion was found in 33% (*N* = 112) of the LR/MWTA group patients. Thirty-three patients (10%) underwent a second surgical treatment due to RHCC, almost half of whom (16) were transplanted. The median time between surgeries amounted to 19.9 months (IQR: 7.3–32.9), and the median waiting list time was 4.4 months (IQR: 1.6–10.3).

The LT group accounted for 222 patients first treated with LT, two of whom received a liver graft from a deceased after-cardiac death donor; the remaining 220 were transplanted with a deceased after-brain death donor liver graft. Out of 222 patients, 177 (80%) were men, and 47 (20%) were women; the median age at the time of transplantation was 59 years (IQR: 53–64) (**Table 1**). The number of Child–Pugh class A patients amounted to 81 (36%), those in class B to 115 (52%), and those in class C to 26 (12%); the median clinical MELD-Na score was 13 (IQR: 10–17) at the time of the LT. On imaging techniques, 130 patients (59%) had monofocal HCC, 52 patients (23%) had bifocal HCC, and 40 patients (18%) had up to three HCC nodules. Native livers were histologically graded as well to moderately differentiated in 132 patients (59%); the remaining 93 (41%) had poorly differentiated HCC. Tumor sizes were histologically classified (pT) as T1 in 90 cases (41%), T2 in 123 cases (55%), and T3 in the remaining nine (4%). The microvascular invasion was found in 43 patients (19%). During the study period, the Italian mean rate of the deceased donation was 21.2 ± 1.8 donors per million inhabitants per year. In contrast, in our region, the mean rate was 10.5 ± 3.2 donors per million per year. In most cases, cirrhosis was secondary to chronic viral infection (66%); in particular, to hepatitis C virus (HCV)-related liver cirrhosis (*n* = 117, 53%), hepatitis B virus (HBV)-related liver cirrhosis (*n* = 29, 13%), alcohol-related liver cirrhosis (*n* = 30, 13%), nonalcoholic fatty liver disease (NASH) (*n* = 42, 19%), and others (*n* = 4, 1.8%).

**Figure 2:**
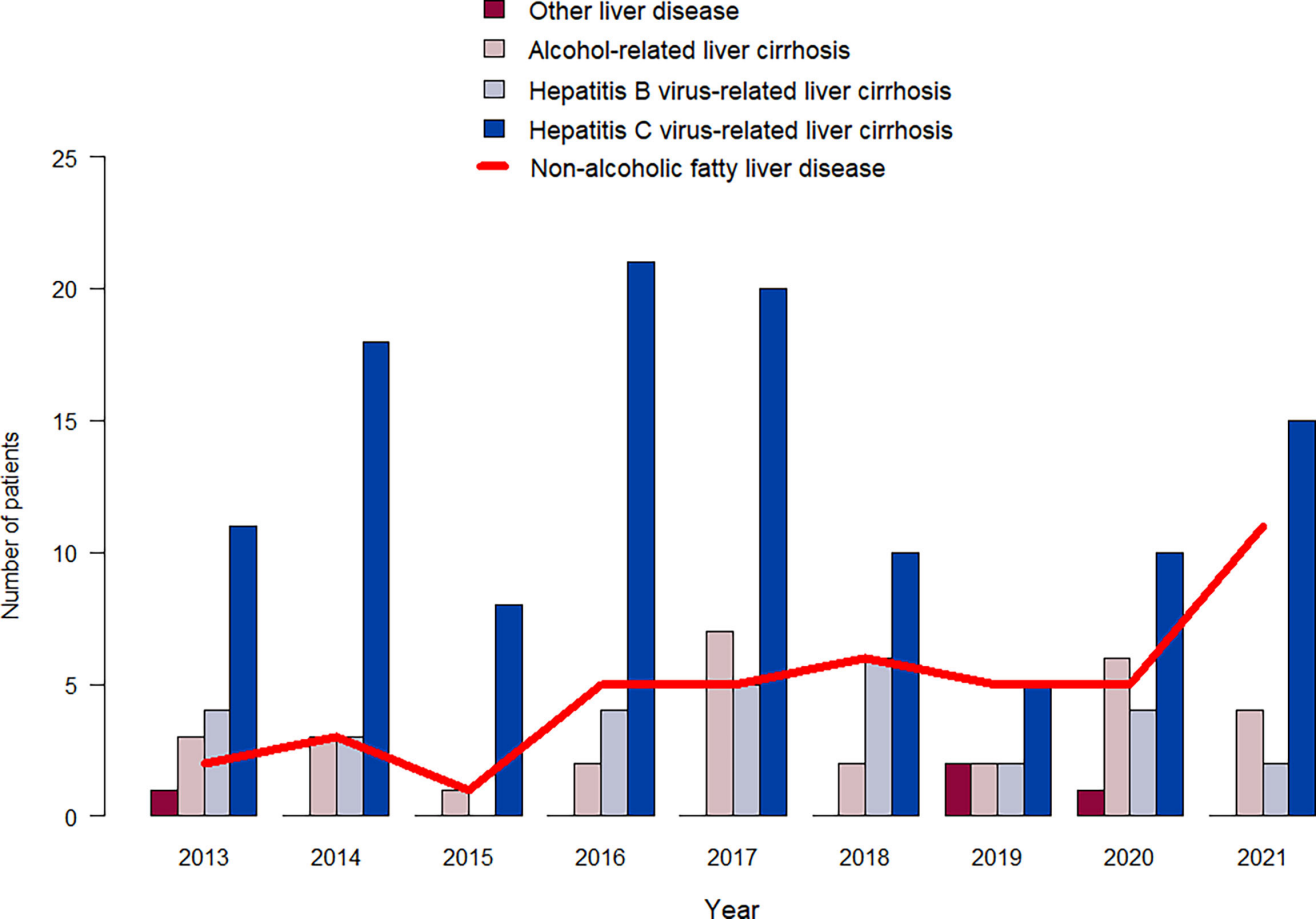
Distribution of different etiologies of liver disease by year of transplantation.

Of note, over the years, there has been an increase in cases of HCC arising from post-NASH cirrhosis (**Figure 2**). The distribution of waiting list times was highly positively skewed with a median of 2.0 months, an IQR of 0.7–6.1, and a range from 0.2 to 133 months. One patient underwent a liver resection due to RHCC 5.7 years after the transplant, and two other patients underwent second liver transplantation due to graft nonfunction (not to RHCC).

### Recurrence of hepatocellular carcinoma

**Figure 3:**
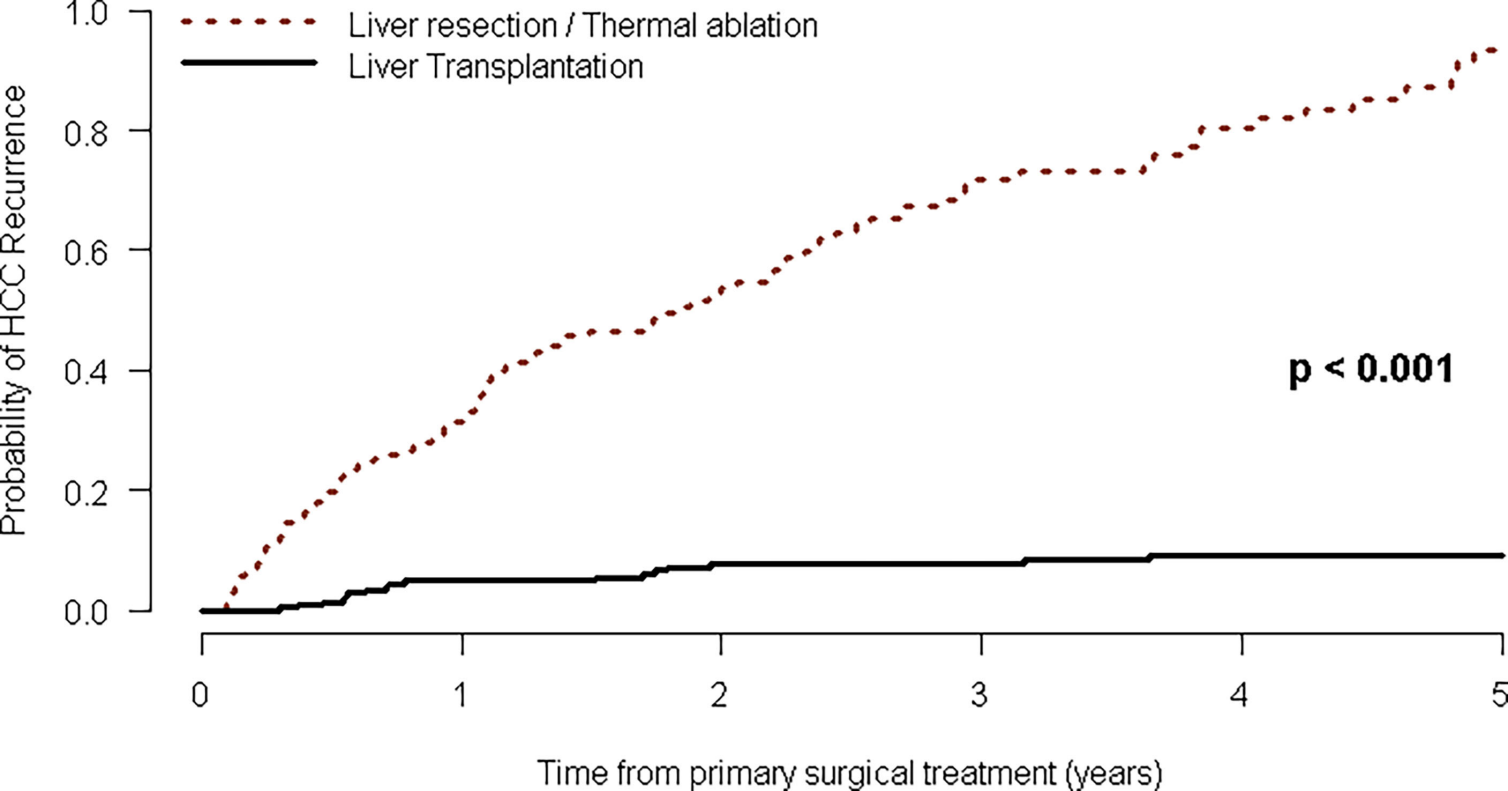
Kaplan–Meier curves of time to HCC recurrence after first-line curative treatment.

The RHCC was markedly higher for patients treated with LR/LRT than for those treated with LT. Overall, 111 patients experienced tumor recurrence in the LR/MWTA group, instead of only 18 transplanted patients. In detail, RHCCs after LT were developed in 18 (8%) out of 222 recipients, of which two patients had only intrahepatic HCC recurrences and the other 16 developed metastases (eight in the lungs, three in the bone, two in the adrenal gland, one in the brain, and two involved multiple extrahepatic systems). After 1, 3, and 5 years from treatment, the estimated recurrence rates for patients in the LR/MWTA group were 32% (95% CI: 24–38), 72% (95% CI: 62–79), and 94% (95% CI: 83–98), respectively, compared to 5% (95% CI: 2–8), 8% (95% CI: 4–12), and 9% (95% CI: 5–14) for patients in the LT groups (**Figure 3**; **Table 2**).

**Table 2:** Hepatocellular carcinoma recurrence rate of 557 patients who underwent surgical treatment.

Time	Liver resection/ablation			Liver transplantation		
	Events	Kaplan–Meier estimate	(95% CI)	Events	Kaplan–Meier estimate	(95% CI)
1 year	55	32%	(24–38)	10	5%	(2–8)
2 years	81	54%	(44–62)	15	8%	(4–12)
3 years	98	72%	(62–79)	15	8%	(4–12)
4 years	104	80%	(70–87)	17	9%	(5–14)
5 years	111	94%	(83–98)	17	9%	(5–14)

Considering the hazard rates (HRs) of the two groups were nonproportional, a univariate Cox model for time to RHCC was fitted in a stratified manner. Within the LR/MWTA group, significantly associated with tumor recurrence were hepatitis B virus infection (which has a protective role with HR: 0.34, 95% CI: 0.13–0.94, *p* = 0.038), number of HCC nodules (HR: 1.54, 95% CI: 1.22–1.94, *p* < 0.001), size of the largest nodule (HR: 1.06, 95% CI: 1.01–1.12, *p* = 0.023), serum bilirubin (HR: 1.57, 95% CI: 1.03–2.40, *p* = 0.038), and international normalized ratio (HR: 16.40, 95% CI: 2.30–118.0, *p* = 0.006). For transplanted patients, the only significant risk factor in univariate analysis was the number of HCC nodules (HR: 1.61, 95% CI: 1.05–2.47, *p* = 0.030) (**Table 3**).

**Table 3:** Cox models for time to hepatocellular carcinoma recurrence after first-line curative treatment.

	Liver resection/thermal ablation			Liver transplantation		
	HR	95% CI	*p*	HR	95% CI	*p*
Male sex	0.94	0.59–1.49	0.802	1.35	0.39–4.66	0.637
Patient’s age	0.99	0.98–1.01	0.468	1.03	0.96–1.10	0.457
Alcohol usage	1.78	0.64–4.92	0.266	0.38	0.05–2.87	0.349
Hepatitis C virus infection	1.40	0.89–2.22	0.148	1.04	0.41–2.63	0.94
Hepatitis B virus infection	**0.34**	**0.13**–**0.94**	**0.038**	1.84	0.60–5.59	0.283
Nonalcoholic fatty liver disease	0.57	0.28–1.18	0.129	0.59	0.14–2.58	0.487
Number of HCC nodules	**1.54**	**1.22**–**1.94**	**<0.001**	**1.61**	**1.05**–**2.47**	**0.030**
Size of the largest nodule	**1.06**	**1.01**–**1.12**	**0.023**	1.36	0.98–1.88	0.064
Serum bilirubin	**1.57**	**1.03**–**2.40**	**0.038**	0.94	0.75–1.17	0.56
International normalized ratio	**16.40**	**2.30**–**118.0**	**0.006**	0.33	0.03–3.36	0.349
Serum creatinine	0.84	0.55–1.28	0.424	0.89	0.35–2.26	0.813
Serum sodium	1.01	0.94–1.08	0.817	1.06	0.93–1.20	0.389
Model for end-stage liver disease	1.03	0.96–1.10	0.384	0.95	0.86–1.05	0.349
Platelets count	1.00	1.00–1.00	0.939	1.00	1.00–1.01	0.25
Microvascular invasion	1.26	0.81–1.96	0.306	0.76	0.08–7.45	0.812
Histological grade ≥3	1.53	0.99–2.36	0.057	1.27	0.18–9.01	0.812
Waiting list time				0.89	0.76–1.05	0.17
Donor age				1.02	0.99–1.05	0.27

The bold values provided information about second treatments, and the following surgical options are the specific treatments:LRMWTACombined LR/MWTAOLT.

**Figure 4:**
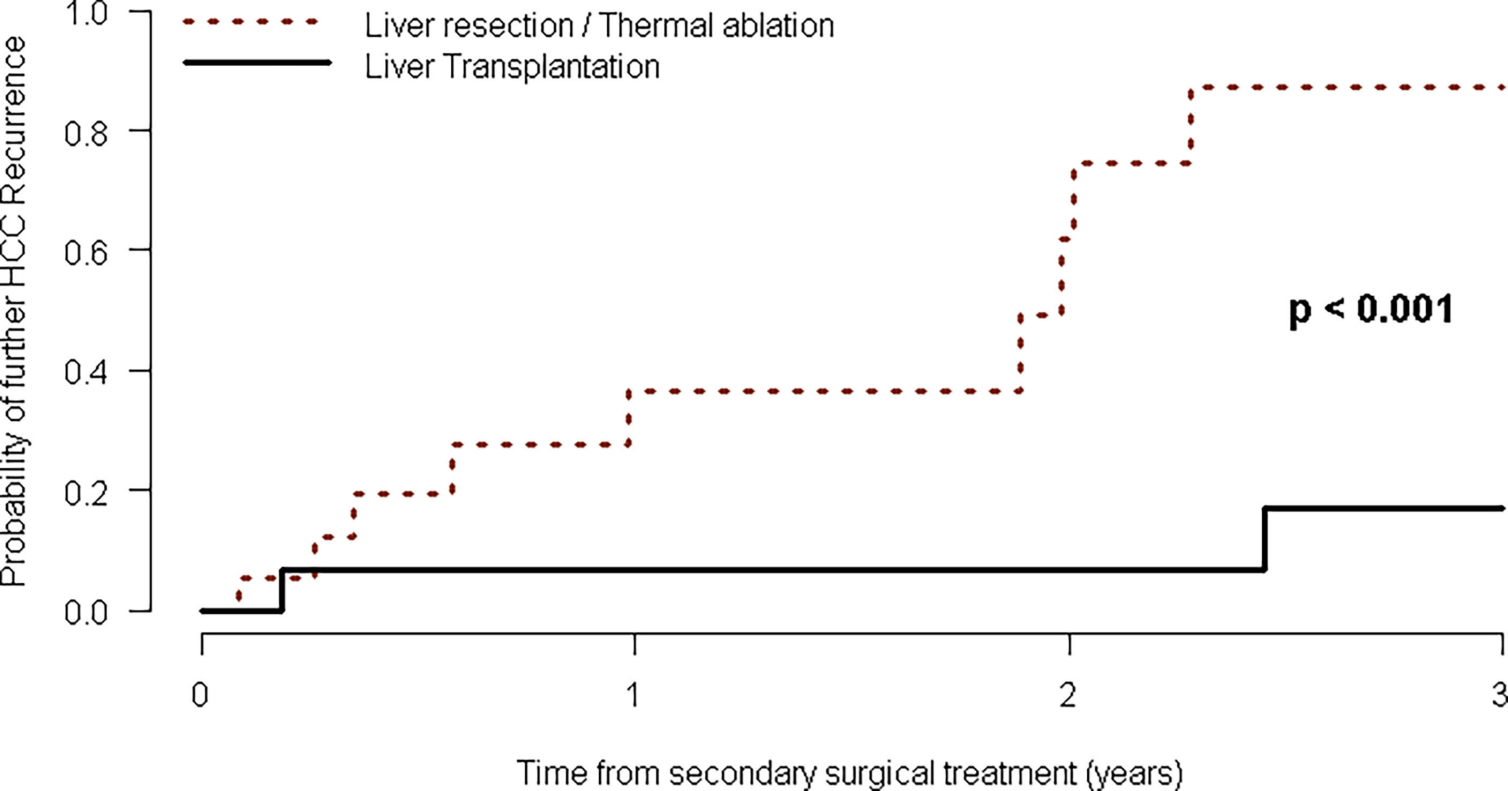
Kaplan–Meier curves of time to HCC recurrence after second-line curative treatment.

Among the 111 patients with RHCC in the first group, 33 were iteratively treated with further curative treatment (12 were treated with LR, two with MWTA, three with a combined LR-MWTA treatment, and 16 underwent LT). Only one of the 18 recurrent patients previously treated with LT underwent LR (**Figure 1**). For these patients who were eligible for liver transplantation as the secondary HCC treatment, 1-, 2-, and 3-year survival were, respectively, 7% (95% CI: 0–19), 7% (95% CI: 0–19), and 17% (95% CI: 0–37), as opposed to those who were treated with LR/MWTA after a previous surgical treatment, whose survival estimates amounted to 37% (95% CI: 4–58), 62% (95% CI: 13–83), and 87% (95% CI: 23–99) at 1, 2, and 3 years, respectively (**Table 4**), thus showing a marked although nonstatistically significant survival experience (log-rank test *p* < 0.001, **Figure 4**).

**Table 4:** Hepatocellular carcinoma recurrence rate of 34 patients who underwent a second surgical treatment.

Time	Liver resection/ablation			Liver transplantation		
	Events	Kaplan–Meier estimate	(95% CI)	Events	Kaplan–Meier estimate	(95% CI)
1 year	5	37%	(4–58)	1	7%	(0–19)
2 years	7	62%	(13–83)	1	7%	(0–19)
3 years	9	87%	(23–99)	2	17%	(0–37)

Uni- and multivariable Cox models were fitted to investigate the risk factors for further RHCC in every 34 patients who were treated with secondary IDLS. In univariate analysis, patient age was found to be a predictor of RHCC in the posttreatment setting (HR: 1.09, 95% CI: 1.01–1.17, *p* = 0.029), instead of the protective roles of LT for the treatment of RHCC after primary IDLS (HR: 0.08, 95% CI: 0.02–0.37, *p* = 0.002), and the impact of previous minimally invasive treatment (HR: 0.24, 95% CI: 0.08–0.76, *p* = 0.015). At multivariable analysis, the best Akaike Information Criterion (AIC) forward stepwise variable selection algorithm confirmed the protective roles of LT for primary RHCC after IDLS (HR: 0.06, 95% CI: 0.01–0.36, *p* = 0.002), of the time relapsed between the first and second IDLS treatments (HR: 0.97, 95% CI: 0.94–0.99, *p* = 0.044), and the impact of previous minimally invasive treatment (HR: 0.28, 95% CI: 0.08–1.00, *p* = 0.051) as the best set of predictors of RHCC, respectively (**Table 5**).

**Table 5:** Cox models for time to hepatocellular carcinoma recurrence after second-line curative treatment.

	Univariable Cox models			Multivariable Cox model		
	HR	95% CI	*p*	HR	95% CI	*p*
Male sex	0.50	0.13–1.95	0.319			
Patient’s age	1.09	1.01–1.17	0.029			
Liver transplantation after first IDLS	0.08	0.02–0.37	0.002	0.06	0.01–0.36	0.002
Time from the first treatment	0.98	0.94–1.02	0.27	0.97	0.94–0.99	0.044
Previous minimally invasive treatment	0.24	0.08–0.76	0.015	0.28	0.08–1.00	0.051

There were 64 deaths (27%) during follow-up. Causes of death were attributable to RHCC in 17 patients (27% of deaths); septic shock and multiorgan failure in 23 patients (36%); liver disease recurrence in three patients (5%); the onset of other (non-HCC) neoplasms in four patients (6%); surgical complication or graft nonfunction in three patients (5%); and the remaining 14 (22% of all dead patients) are attributable to other causes, such as cardiac arrest, cerebral hemorrhage, and fulminant meningitis–encephalitis. Overall survival estimates at 1, 3, and 5 years after LT were, respectively, 87% (95% CI: 83–91), 73% (95% CI: 68–80), and 68% (95% CI: 62–75).

## Discussion

The non-HCC field for hepato-pancreato-biliary surgeons has expanded in both scope and surgical indications (31, 32). There are different treatment options for HCC concerning liver function and the type of tumor. The EASL-EORTC guidelines recommend hepatectomy for HCC patients at BCLC stage 0 or BCLC-A with no portal hypertension (PHT) (4). Indication for surgery also depends on the number of nodules, the diameter of tumors, vascular invasion, and extrahepatic disease. In the 2016 EASL updated guidelines, liver resection was also introduced as a possible treatment for patients with PHT by endorsing a risk algorithm for postoperative liver decompensation. This algorithm included the MELD score, the presence of PHT, and type of resection (6).

A recent multicentric study showed that cirrhotic patients with a hepatic venous pressure gradient of ten or more could undergo liver resection with an acceptable 90-day perioperative mortality and morbidity (6% and 27%, respectively) and persistent liver decompensation (10% at 3 months) (7). In many Asian studies, the extent of surgery was applied where technically feasible, including in patients with macrovascular invasion (33, 34). In 2016, a study conducted on 6,474 patients affected by HCC and macrovascular invasion compared surgical vs. nonsurgical treatment demonstrated a median and 5-year survival of 29.4 months and 32.9% in the resected patients versus 18.8 months and 20.1% in patients treated nonoperatively (35).

Nevertheless, for successful outcomes after LRT, LT represents the only valid treatment for both malignancy and underlying cirrhosis. Among our transplanted patients within the Milan criteria, the HCC recurrence rate is about 9%, confirming data existing in the literature (36, 37).

In this study, the maximum nodule size, number of nodules, serum bilirubin, and international normalized ratio represent risk factors for RHCC after LRT. In the same way, some studies in the literature have also demonstrated the role of end-stage liver disease in RHCC. In this setting, precise scores, such as the Model of Recurrence After Liver transplant score, have yet to provide a specific tool for predicting RHCC and risk stratification pre- and postoperatively (38).

Several studies have focused their interest on finding the best model to predict post-LT HCC recurrence. Firstly, the Milan criteria in 1996 included tumor burden and the number of nodules at explant (11). In 2012, a multicentre French study incorporated an AFP threshold, the number of nodules, and the largest tumor diameter into a prognostic score (36). In our multivariate analysis, we confirmed the prognostic role of and a number of nodules, and these data are widely validated in the literature; the RETREAT score showed elevated AFP, the presence of microvascular invasion on the explant, and the largest viable tumor diameter plus the number of viable tumors on the explant, as possible prognostic factors (37).

However, in cases of patients beyond the Milan criteria, the recent XXL trial showed that effective downstaging treatment correlates significantly with a higher tumor event-free survival after LT (*p* = 0.003) (10, 39, 40). The United Network of Organ Sharing (UNOS) guidelines suggest a downstaging protocol for patients beyond MC, focusing on their response to bridge therapy (41). Also, a recent study demonstrated that disease progression after bridging therapy is an independent risk factor for recurrence and mortality (42).

Another relevant data emerging from our multivariate analysis is the “protective role” of the minimally invasive approach. If patients develop RHCC much more time after the first IDLS, it is possible to experience longer tumor-free survival even after subsequent surgical treatments (42–46). The clinical entity of RHCC can be developed in different settings, and it depends on which first-line therapy was chosen. There is little information in the literature about this type of patient because those who underwent an intentional curative surgical treatment are not initially evaluated for liver transplantation. These data also strengthen the importance of tumor behavior and surveillance for this group of patients.

Recently, in the first prospective, randomized, controlled trial, there has been evidence of longer patient survival and fewer tumor events after LT in patients who achieved success and sustained downstaging of HCCs exceeding the Milan criteria, compared with those in the nontransplantation therapy group (10). In this retrospective study, we showed the protective roles of LT for primary RHCC after IDLS and of the time relapsed between the first and second IDLS treatments for patients affected by HCC with more advanced liver disease and higher bilirubin levels. In this setting, the impact of previous minimally invasive treatment could lead to lower rates of HCC recurrence and peritoneal adhesions On the other hand, this study has different limitations: the retrospective design, the inclusion only of patients within MC, and the selection of patients referred to a liver transplant center.

For our transplant center, considering the low rate of deceased donation, we can suggest that IDLS represents the best option for patients affected by HCC and fit for surgery, but these results confirm the importance of starting the evaluation for LT both for recurrence and mortality.

## Data availability statement

The raw data supporting the conclusions of this article will be made available by the authors, without undue reservation.

## Ethics statement

The studies involving human participants were reviewed and approved by ISMETT Ethics Committee. The patients/participants provided their written informed consent to participate in this study.

## Author contributions

Made substantial contributions to conception and design of the study and performed data analysis and interpretation: DP, MB, DC, FF, SL, and SG. Made substantial contributions to data interpretation: PB, SC, GD, CC, MC, and SG. Performed data acquisition, as well as providing administrative, technical, and material support: DP, SK, BM, and SG. All authors listed have made a substantial, direct, and intellectual contribution to the work and approved it for publication.

## Funding

This work was supported by the Italian Ministry of Health, Rome, Italy (Ricerca Corrente: RC 2022, Linea 1E).

## Conflict of interest

The authors declare that the research was conducted in the absence of any commercial or financial relationships that could be construed as a potential conflict of interest.

## Publisher’s note

All claims expressed in this article are solely those of the authors and do not necessarily represent those of their affiliated organizations, or those of the publisher, the editors and the reviewers. Any product that may be evaluated in this article, or claim that may be made by its manufacturer, is not guaranteed or endorsed by the publisher.
